# Systematic review: genotypic and phenotypic resistance of fluoroquinolone-resistant *Salmonella* in livestock in South America (2020–2024)

**DOI:** 10.3389/fvets.2025.1614486

**Published:** 2025-09-03

**Authors:** Stefany Barrientos-Villegas, María Isabel García-Álvarez, Juana L. Vidal, Luis M. Gómez-Osorio, Sara López-Osorio, Jenny J. Chaparro-Gutiérrez

**Affiliations:** CIVAB Research Group, Faculty of Agricultural Sciences, University of Antioquia (UdeA), Medellín, Colombia

**Keywords:** quinolones, fluoroquinolones, antimicrobial resistance, *Salmonella*, South America

## Abstract

**Objective:**

To determine the frequency of phenotypic and genotypic resistance to quinolones and fluoroquinolones in *Salmonella* spp. isolated from production animals (pigs, poultry, cattle) and rodents in South America between 2020 and 2024, with the goal of providing key information on resistance in these countries for public health and food safety.

**Methods:**

A systematic review was conducted following the PRISMA guidelines, using databases such as Scopus, PubMed, SciELO, and Latindex. Studies on *Salmonella* spp. resistant to quinolones and fluoroquinolones in production animals, meat products, and rodents in South America during 2020–2024 were included.

**Results:**

Of the 83 initial results, 27 studies were selected. 70.4% of the studies were conducted in Brazil. 88% of the studies (*n* = 24/27) used phenotypic methods, with the disk diffusion technique being the most common. Ciprofloxacin was the most studied antibiotic, with an overall resistance of 32.5%, followed by nalidixic acid (60.6%) and enrofloxacin (23.7%). The average multidrug resistance (MDR) was 62%. 44% of the studies (*n* = 12/27) employed genotypic methods, with whole genome sequencing (WGS) being the most notable technique. Mutations were reported in *parC* (58%), *gyrA* (50%), *gyrB* (8%), and the presence of *qnr* genes (75%) and *aac(6′)-Ib-cr* (8%). No studies on rodents were found.

**Conclusion:**

Resistance to quinolones and fluoroquinolones in *Salmonella* spp. in South America endangers public health and food safety. To address antimicrobial resistance, monitoring and control measures must be implemented, regional research should be promoted, and stronger restrictions should be enforced.

## 1 Introduction

*Salmonella* spp. belongs to the Enterobacteriaceae family, with over 2,600 serotypes reported, affecting a wide range of animals, including humans (1). The *Salmonella* genus consists of two species: *enterica* and *bongori* (2). *S. enterica* is classified into six subspecies: *enterica* (subsp. I), *salamae* (subsp. II), *arizonae* (subsp. IIIa), *diarizonae* (subsp. IIIb), *houtenae* (subsp. IV), and *indica* (subsp. VI). Subspecies I is associated with more than 99% of the diseases caused by *Salmonella* in warm-blooded animals, including gastroenteritis and enteric fever (3).

Nontyphoidal salmonellosis has been the most commonly reported zoonotic disease in humans (4). It is acquired through three main routes: food, animal handling on farms or carcass handling in slaughterhouses, contact with pets (dogs and cats), and exotic animals (birds, ferrets, lagomorphs, mustelids, reptiles, and rodents) (5). *Salmonella* transmission has been primarily linked to contaminated water and food sources, including eggs, meats, and vegetables (6). In this context, the prevention and control of pathogens are ongoing challenges, which is why antimicrobials are frequently used in veterinary medicine to treat and prevent diseases (7). However, there is growing concern that the use of these in animal production may compromise human health through the zoonotic transfer of resistant bacteria via contaminated animal-derived food, direct contact, and their spread in the environment (8).

Nontyphoidal salmonellosis mainly causes self-limiting gastroenteritis in both humans and animals (9). However, when this infection becomes invasive, it requires antibiotic treatment. If the pathogens show resistance, it limits the therapeutic options available for the patient (10). Fluoroquinolones (FQ) have been widely used in clinical practice for the treatment of salmonellosis in both humans and animals (11) and Ciprofloxacin is the first-line antibiotic used to treat both typhoidal and nontyphoidal salmonellosis in humans (9, 12). However, the emergence of resistance or multidrug resistance (MDR) to these antibiotics has become a critical issue in the clinical treatment of the disease (13). This is why the World Health Organization (WHO) classifies fluoroquinolone-resistant *Salmonella* as a high-priority pathogen to support research and the development of new antibiotics (12, 14).

Resistance to fluoroquinolones in *Salmonella* can occur due to mutations in the quinolone resistance-determining regions (QRDR) of the chromosomal *gyr* and *par* genes, resulting in a reduced binding affinity of the topoisomerase enzymes to quinolones (15). Secondly, plasmid-mediated quinolone resistance (PMQR) involves the acquisition of (i) *qnr* genes *(qnrA, qnrB, qnrS, qnrC, qnrD),* which encode topoisomerase-binding proteins that provide physical protection against quinolones, (ii) the *aac(6′)-Ib-cr* gene, which encodes a modifying enzyme that reduces the activity of fluoroquinolones, and (iii) *oqxAB* and *qepA*, which encode quinolone efflux pumps. Finally, the negative and positive regulation of porins encoded by chromosomal genes or the efflux pumps of multiple drugs (AcrAB-TolC), respectively, reduce intracellular concentrations of fluoroquinolones (14).

In South America, the dynamics of antimicrobial resistance (AMR) in *Salmonella* within the animal sector—particularly in production animals and across the food supply chain—are poorly characterized. Countries such as Venezuela, Guyana, French Guiana, and Suriname reported very limited research between 2020 and 2024, highlighting significant gaps in AMR surveillance and data. Understanding the current status of fluoroquinolone-resistant *Salmonella* is especially critical, given the scarcity and fragmentation of existing evidence. This study aims to consolidate available data on phenotypic and genotypic resistance to quinolones and fluoroquinolones in *Salmonella* isolated from pigs, poultry, cattle, and rodents. The inclusion of rodents is supported by their established role as reservoirs and amplifiers of zoonotic pathogens in agricultural environments. By providing a unified analysis, this review contributes to a clearer understanding of the regional AMR landscape and supports the development of targeted public health strategies, including improved biosecurity and responsible antibiotic use on farms.

## 2 Materials and methods

### 2.1 Study search

The study was conducted following the guidelines established in the Preferred Reporting Items for Systematic Reviews and Meta-Analyses (PRISMA)(16). The study populations included *Salmonella* isolates from production animals such as cattle, poultry, pigs, meat products from these animals, and rodents. The primary outcome of interest was the reported frequencies of phenotypic and genotypic resistance to FQ: non-susceptibility to nalidixic acid (Nal ns), non-susceptibility to ciprofloxacin (Cip ns), non-susceptibility to enrofloxacin (Enr ns), frequency of mutations in QRDR genes, and the presence or absence of PMQR genes. The secondary outcomes included MDR, reported serotypes and sequence types (ST), amino acid substitutions in mutated genes, and the phenotypic and genotypic techniques used. MDR was defined as resistance to three or more drugs.

A literature search was conducted in English, Spanish, Portuguese, and French using Boolean logic tools with the operators “AND” and “OR” to search for relevant articles in the PubMed, Scopus, SciELO, and Latindex databases. The search aimed to identify pertinent articles published from January 1, 2020, to August 24, 2024. The search string that allowed for the identification of most studies was as follows: *Salmonella* AND (quinolone OR fluoroquinolone OR ciprofloxacin OR nalidixic acid OR enrofloxacin) AND (livestock OR cattle OR swine OR pig OR poultry OR rodent OR rat OR beef OR chicken OR pork OR meat) AND (Peru OR Brazil OR Colombia OR Ecuador OR Chile OR Venezuela OR Argentina OR Uruguay OR Bolivia OR Guyana OR Paraguay OR French Guiana OR Suriname). The search was conducted on August 24, 2024. Additional articles were also included, manually located in the Scopus, PubMed, SciELO, and Latindex databases. Additional articles were also included through manual searches of reference lists from selected studies and relevant journals.

### 2.2 Study selection

The study selection was carried out by two independent reviewers (SBV and MIG), and the references were exported to the Rayyan online application software for screening and selection. In the first phase of review (screening), titles and abstracts were evaluated to identify studies related to the primary outcomes of interest. At this stage, exclusion criteria were applied to discard studies whose titles and/or abstracts were not relevant. Discrepancies between reviewers were resolved through discussion or, if necessary, by consulting a third reviewer.

In the second phase (eligibility), a full-text review of the selected articles was conducted, with detailed assessment based on the following eligibility criteria: (i) publication in English, Spanish, Portuguese, or French; (ii) inclusion of phenotypic and/or genotypic determinants of fluoroquinolone resistance; (iii) isolation of *Salmonella* from production animals, meat products, or rodents; (iv) exclusion of incomplete or unclear studies; (v) exclusion of studies conducted outside South America; and (vi) inclusion of studies published before August 24, 2024.

Duplicate references were identified and removed using EndNote software prior to the screening process. Additionally, data extraction was performed by one reviewer and independently validated by a second reviewer to minimize errors or inconsistencies. To assess the risk of bias and methodological quality of the included studies, we used the Joanna Briggs Institute (JBI) Critical Appraisal Checklist for prevalence studies. Two independent reviewers (SBV and MIG) performed the quality assessment, and disagreements were resolved by consensus or by involving a third reviewer. Studies were not excluded based on quality, but the appraisal results were considered when interpreting the findings.

### 2.3 Data extraction

The following data were considered and extracted: (i) study identifier: Title, authors, year of publication, country, species (production animals, meat animals, or rodents); (ii) Methods: sample type, sample size, identification method, antimicrobial susceptibility testing (fluoroquinolones or quinolones), breakpoint/interpretive standard level, and gene detection (phenotype-based/genotype-based); (iii) Results: number of isolates, number of isolates tested for susceptibility, number of MDR strains, number of strains resistant to nalidixic acid (Nal ns), number of strains resistant to ciprofloxacin (Cip ns), number of strains resistant to enrofloxacin (Enr ns), number of strains examined for mutation detection (*gyrA, gyrB, parC* y *parE*), number of mutants, mutation positions, substituted amino acids, number of strains examined for plasmid-mediated quinolone resistance genes (PMQR) (*qnrA, qnrB, qnrC, qnrD, qnrS, aac(6′)-Ib-cr, qepA, oqxA/B*) and number of strains with PMQR genes.

### 2.4 Data analysis

Data were analyzed using descriptive statistics in Microsoft Excel (Microsoft 365®). Additionally, GIS software (QGIS 3.16.15) was used to generate maps illustrating the distribution of resistance patterns across South America.

Inferential statistical analyses were not applied in this study due to the high heterogeneity among the included articles in terms of study design, sample sizes, animal species, sampling matrices, and laboratory methodologies. As a result, quantitative synthesis through meta-analysis was not feasible. A descriptive approach was used instead, in line with the exploratory nature of this review.

## 3 Results

**Figure 1:**
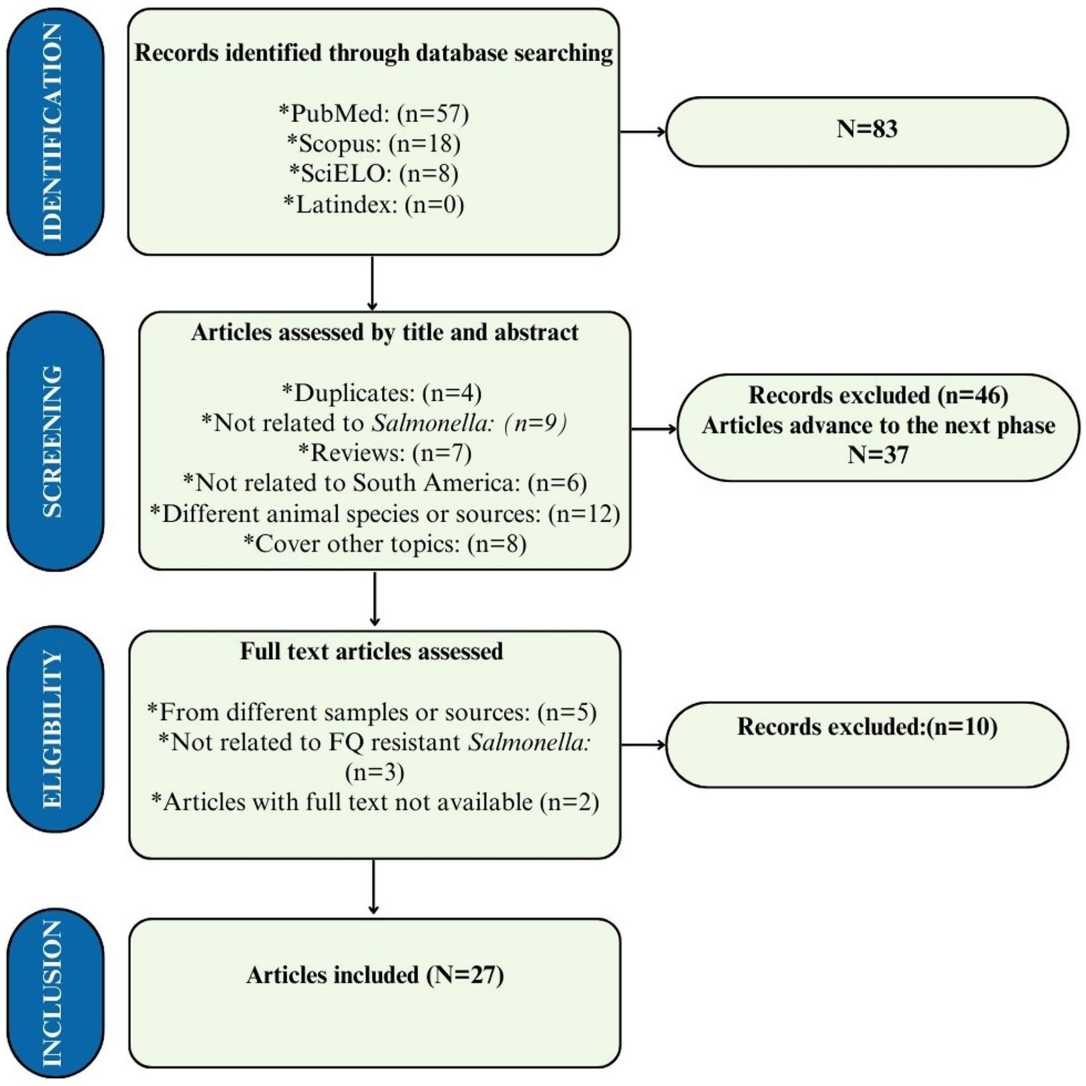
PRISMA flow diagram for study categorization and selection of the 27 studies included in this systematic review. Data came from databases between 2020 and 2024.

Our search strategy yielded a total of 83 results, with 57 found in PubMed, 18 in Scopus, and 8 in SciELO. After excluding 46 articles based on their title and abstract, 37 were selected for full-text reading, and of these, only 10 were excluded, resulting in a total of 27 articles included in this study. The main reason for excluding articles during the selection process was that they did not analyze the *Salmonella* agent or did not focus on relevant animal matrices of interest (Figure 1).

According to our results, 70.4% (*n* = 19/27) of the studies were conducted in Brazil, followed by Argentina and Colombia with 7.4% (*n* = 2/27) each. Next, Paraguay, Uruguay, Chile, and Ecuador each represented 3.7% (*n* = 1/27).

Regarding the species studied, 40.74% of the articles focused on swine and its products (*n* = 11/27), a percentage equal to that of poultry and its products (*n* = 11/27). Cattle and their products were investigated in 3.7% (*n* = 1/27), while 14% (*n* = 4/27) involved a combination of matrices from different species (swine, cattle, and poultry). No published research was found regarding rodents.

### 3.1 Phenotypic resistance to quinolone and fluoroquinolone in *Salmonella*

Of the 27 studies, 88% (*n* = 24/27) employed phenotypic methods to detect fluoroquinolone resistance in *Salmonella* isolates the most commonly method used to assess AMR was disk diffusion (17), in 75% (*n* = 18/24) of the studies, followed by broth microdilution (18) in 25% (*n* = 6/24) of the studies, according to CLSI guidelines. The most studied fluoroquinolone was ciprofloxacin, present in 91.6% (*n* = 22/24) of the studies, with an overall resistance of 32.53%. It was followed by nalidixic acid, studied in 15 of the 24 studies, which showed an overall resistance of 60.6%. In contrast, enrofloxacin was the least evaluated, being analyzed in 10 of the 24 studies, with an overall resistance of 23.74%. MDR was assessed in 17 of the 24 studies (*n* = 17/24), with an overall percentage of 62%.

#### 3.1.1 Poultry isolates

The most used sample type in poultry studies was a combination of matrices from various sources, accounting for 37.5% (*n* = 3/8), followed by feces and chicken meat. The most frequently isolated serotype was *Salmonella* Heidelberg, reported in 62.5% of the studies (*n* = 5/8), with average resistances in chicken meat of 96.3, 38.9% to nalidixic acid and ciprofloxacin, respectively. In feces, resistances of 54% to ciprofloxacin were found (Table 1).

**Table 1:** Phenotypic resistance to FQ into *Salmonella* in poultry isolates in South America (2020–2024).

Author	Country	*Salmonella* isolation	Sample type	Serotypes	Nal ns		Cip ns		Enr ns		MDR
Souza et al. (26)	Brazil	*n* = 62	Cloacal swabs, drag swabs, feeders, drinkers and Poultry meat	*S*. Heidelberg	Meat 20/20 Poultry 9/10 Farm 21/32	(80.65%)	Meat *n* = 0/20 Poultry *n* = 0/10 Farm *n* = 1/32	(1.61%)	Meat *n* = 4/20 Poultry *n* = 2/10 Farm *n* = 12/32	(29.03%)	41/62
Lucca et al. (30)		*n* = 22	Carcasses broilers	*S*. Pullorum, *S. H*eidelberg *S. C*orvalis	-	-	18/22	(81.82%)	2/22	(9.09%)	14/22
Monte et al. (32)		*n* = 108	Poultry production: Surfaces Transport Processing Final product	*S. Enteritidis*	-	-	1/108	(0.93%)	20/108	(18.52%)	-
Perin et al. (28)		98/300	Chicken meat	*S. T*yphimurium, *S*. Heidelberg, *S*. Ndolo, *S*. Minnesota, 0:4,5, *S*. Thompson, *S*. Schwarzengrund, *S*. Abani, o:3,10:e,h	93/98	(94.90%)	74/78	(75.51%)	-	-	84/98
Moreira et al. (25)		*n* = 25	Poultry feces	*S*. Minnesota	7/25	(28%)	4/25	(16%)	-	-	16/25
Grossi et al. (31)		*n* = 96	Chicken carcasses, bird cages/transport boxes,and end cuts	*S.* Heidelberg, *S*. Schwarzengrund, *S*. Anatum, O:4,5	-	-	2/96	(2.08%)	-	-	6/96
Herrera-Sánchez et al. (24)	Colombia	*n* = 39	Broiler feces	*S*. Heidelberg, *S. paratyphi* B.	-	-	36/39	(92.31%)	19/39	(48.72%)	-
Lapierre et al. (27)	Chile	87/360	Chicken meat	*S. Infantis*	85/87	(97,70%)	2/87	(2.30%)	3/87	(3.45%)	82/87

Phenotypic resistance in *Salmonella*, broken down by country and matrix. The ex: *n* = 65 in the “*Salmonella* Isolation” column refers to previously isolated strains, while the fractions indicate the proportion of the agent isolated in the analyzed samples. The symbol “–” denotes concepts not studied in that specific analysis, allowing for easy identification of areas lacking information.

#### 3.1.2 Pigs isolates

The most commonly used sample type in swine studies was a combination of matrices from various sources, representing 45% (*n* = 5/11) of the cases. The most frequently reported serotype was *S. typhimurium*, found in 81.8% (*n* = 9/11) of the isolates, followed by *S*. Derby, which appeared in 63.6% (*n* = 7/11). On average, in the mesenteric lymph nodes, the most studied individual matrix, resistances of 44, 40, and 37% were observed for nalidixic acid, ciprofloxacin, and enrofloxacin, respectively (Table 2).

**Table 2:** Phenotypic resistance to FQ into *Salmonella* in pig isolates in South America (2020–2024).

Author	Country	*Salmonella* isolation	Sample type	Serotypes	Nal ns		Cip ns		Enr ns		MDR
Azevedo et al. (42)	Brazil	29/100	Porcine mesenteric lymph nodes	*S*. Derby. *S.* Cerro, *S*. Give	19/29	65.5%	23/29	79.3%	16/23	69.6%	23/29
de Quadros et al. (63)		19/90	Pig carcass swabs	*S*. Typhimurium, *S*. Derby, *S*. Infantis	-	-	-	-	*n* = 0/90	0%	9/25
Simoni et al. (46)		*n* = 140	Environment, pig carcass, lymph nodes, intestinal content, and pork	*S.* Derby isolates collected over a 10-year	13/140	9.29%	0/140	0%	-	-	-
Viana et al. (54)		*n* = 41	Swines samples from lairage, barn floors, mesenteric lymph nodes, tonsils, swine carcasses and knives	*S*. Typhimurium S. I 4,[5],12:i:- *S. B*redeney *S*. Brandeburgo *S*. Panama *S*. Londres *S*. Mbandaka *S*. Derby *S*. Bovismorbificans	-	-	21/41	51.22%	-	-	30/41
Possebon et al. (43)		91/250	Swine mesenteric lymph nodes	*S*. Typhimurium, *S*. I.4,5,12:i:- *S*. Infantis y *S*. Havana	37/91	40.66%	-	-	-	-	64/91
Pissetti et al. (45)		*n* = 413	Intestinal swine content, lymph nodes, carcasses and products of swine origin.	*S*. Typhimurium *S*. Derby isolates from 2000 to 2015	-	-	77/413	18,64%	-	-	-
Cabral et al. (52)		*n* = 29	Intestinal faeces, mesenteric and submandibular lymph nodes, jowl, ham and from the water for cleaning the carcasses in swine slaughterhouses	*S*. Typhimurium	19/29	65.52%	19/29	0.41%	14/29	48.28%	17/29
Kich et al. (64)		65/378	Pig carcasses	*S*. Typhimurium, *S. infantis*	28/61	45.90%	1/61	1.6%	-	-	32/61
Vidal et al. (67)	Colombia	149/653	Pigs fecal samples	*S*. Typhimurium, S.I,4,12:i:–, *S*. Enteritidis, *S*. Virchow, *S*. Bovismorbificans, *S. E*dinburg, *S*. Heidelberg, *S*. Infantis, *S*. Manhattan,	-	-	76/139	54.68%	-	-	61/139
Parada et al. (38)	Argentina	*n* = 55	Organs, feces and mesenteric nodes from pigs	*S*. Anatum, Brandenburg, Bredeney, Choleraesuis, Derby, Glostrup, Heidelberg, Infantis, Livingstone, Montevideo, Oranienburg, Panama, Rissen, Typhimurium.	*n* = 29/55	52.73%	10/55	18.18%	-	-	31/55
Vico et al. (66)		241/580	Swine mesenteric lymph nodes	*S*. Anatum, Typhimurium, Panama, I 1,3,19: Z10:-, I 4,5,12: I:-, 4,5,12:d:-, Lexington, Westhampton, Derby, Adelaide, Bredeney, Corvallis, Javiana, Minnesota, Mbandaka	13/50	26.00%	0/50	0.00%	2/50	4%	43/50

Phenotypic resistance in *Salmonella*, broken down by country and matrix. The ex: *n* = 65 in the “*Salmonella* Isolation” column refers to previously isolated strains, while the fractions indicate the proportion of the agent isolated in the analyzed samples. The symbol “–” denotes concepts not studied in that specific analysis, allowing for easy identification of areas lacking information.

#### 3.1.3 Cattle isolates

According to a study conducted in Uruguay isolates with non-susceptibility to fluoroquinolones were reported, with 77.3% (*n* = 58/75) of the isolates being non-susceptible to ciprofloxacin, which is a second-generation fluoroquinolone. Additionally, in this group, 6.6% (*n* = 5/75) were non-susceptible to enrofloxacin. 56% (*n* = 42/75) were MDR (Table 3).

**Table 3:** Phenotypic resistance to FQ *Salmonella* in cattle isolates in South America (2020–2024).

Author	Country	*Salmonella* isolation	Sample type	Serotypes	Nal ns		Cip ns		Enr ns		MDR
Casaux et al. (41)	Uruguay	*n* = 75	Calves, cows, heifer, organs, samples from environment, food sample, udder swab, drinking water, bovine fetus autopsy.	*S*. Typhimurium, *S*. Newport, *S*. Anatum, *S*. Dublin, *S*. Agona, *S*. Montevideo y IIIb 61:i:z53	-	-	58/75	77.33%	5/75	6.67%	42/75

Phenotypic resistance in *Salmonella*, broken down by country and matrix. The ex: *n* = 65 in the “*Salmonella* Isolation” column refers to previously isolated strains, while the fractions indicate the proportion of the agent isolated in the analyzed samples. The symbol “–” denotes concepts not studied in that specific analysis, allowing for easy identification of areas lacking information.

#### 3.1.4 Combined matrices

Of the 24 studies that used phenotypic methods, 4 of them used matrices involving a mixture of different species, primarily from their meat products. The average resistance to nalidixic acid was 75%, and to ciprofloxacin was 35% (Table 4).

**Table 4:** Phenotypic resistance in *Salmonella* in combined matrices in South America (2020–2024).

Author	Country	*Salmonella* isolation	Sample type	Serotypes	Nal ns		Cip ns		Enr ns		MDR
Ortiz et al. (65)	Paraguay	*n* = 98	Food animals	*S.* Heidelberg, *S*. Tennessee, *S*. Anatum	80/98	81.63%	1/98	1%	-	-	-
Gomes et al. (48)	Brazil	57/780	Chicken and Pork	*S. H*eidelberg, *S*. Typhimurium and Give, *S*. Schwarzengrund	Chicken 48/58 Pork *n* = 23/60	60.17%	Chicken 43/58 Pork *n* = 18/60	51.69%	-	-	Chicken 46/58 Pork *n* = 30/60
Vilela et al. (68)		*n* = 5	Swine gallbladder, Chicken spleen, gallbladder and illeum	*S. choleraesuis* isolates	3/5	60%	4/5	80%	-	-	-
Vilela et al. (29)		*n* = 11	Chicken meat, bovine meat, animal feed, and a drag swab.	*S*. Heidelberg	11/11	100%	1/11	9%	-	-	-

Phenotypic resistance in *Salmonella* spp. broken down by country and matrix. The ex: *n* = 65 in the “*Salmonella* Isolation” column refers to previously isolated strains, while the fractions indicate the proportion of the agent isolated in the analyzed samples. The symbol “–” denotes concepts not studied in that specific analysis, allowing for easy identification of areas lacking information.

### 3.2 Genotypic resistance to quinolone and fluoroquinolone in *Salmonella*

Of the 27 studies reviewed, 44% (*n* = 12/27) employed genotypic techniques to detect resistance genes in *Salmonella*. The most commonly used methodology was WGS, with the Illumina MiSeq platform being the most widely used, employed in 71% (*n* = 5/7) of the studies that applied Whole Genome Sequencing (WGS), followed by Illumina HiSeq. In comparison, the PCR technique was used in a smaller number of studies, as shown below. In all of the studies that used these techniques, mutations were found in *gyrA* 50% (*n* = 6/12), *parC* 58% (*n* = 7/12), *gyrB* 8% (*n* = 1/12), *qnr* genes 75% (*n* = 9/12), and *aac(6′)-Ib-cr* 8% (*n* = 1/12).

#### 3.2.1 Polymerase chain reaction (PCR) techniques

PCR involves DNA extraction, amplification with specific primers, and thermal cycling, followed by detection via gel electrophoresis or real-time PCR (19). This rapid and sensitive technique enables precise detection of microorganisms and resistance genes, even in low-DNA samples (20). Of the 27 studies, only 19% (*n* = 5/27) used PCR techniques or molecular identification of resistance genes, and all of them correspond to different South American countries. Among the most commonly investigated genes are PMQR genes, highlighting *qnrB*, which was present in 100% of the studies that employed molecular methods, where the most commonly used primer sequences were: qnrb-F GATCGTGAAAGCCAGAAAGG and qnrb-R ACGATGCCTGGTAGTTGTCC (21).

In two of these five studies, QRDRs (quinolone resistance-determining regions) genes were identified, which are chromosomal and result from mutations in the *gyr* and *par* genes. Of these mutations, the most frequently investigated was in the *gyrA* gene, which was analyzed in two of the five studies that used these techniques and primarily presented mutations at position 83. The *parC* gene was only investigated in one study, where mutations T57S were found (Table 5).

**Table 5:** Genes conferring resistance to quinolones and fluoroquinolones identified in *Salmonella* spp. using PCR techniques in South America between 2020 and 2024.

Author	Country	Type of samples	Number of samples	Target genes	Number of mutations	Point mutations (QRDR)
Parada et al. (38)	Argentina	Organs, feces and mesenteric nodes from pigs	30	*Amino acid substitutions of QRDRs: ***gyrA*** *PMQR: ***qnrB***	30/30 had a ***gyrA*** mutation 16/30 had a ***qnrB*** gene	***gyrA:*** (S83Y, S83F, D87G, (S83Y + D72E))
Herrera-Sánchez et al. (24)	Colombia	Broiler feces	39	*PMQR: ***qnrA,B,C, D,S***	24/39 had a **qnrB** gene 1/39 ***aac(6′)-Ib-cr*** gen	-
Lapierre et al. (27)	Chile	Chicken meat	87	*PMQR*: **qnrB***	2/3 had a ***qnrB*** gene	-
Grossi et al. (31)	Brazil	Chicken carcasses, bird cages/transport boxes,and end cuts	96	*Amino acid substitutions of QRDRs:*gyrA* and *parC* *PMQR: ***qnrB,S***	0/96 had a ***gyrA*** mutation 96/96 had a ***parC*** mutation 94/96 had a ***qnrB*** gene 0/96 had a ***qnrS*** gene	***parC***: (T57S)
Ortiz et al. (65)	Paraguay	Food animals	41	*PMQR:***qnrA,B,S***	13/41 had a ***qnrB*** gene 1/41 had ***qnrsB+qnrS*** genes	-

(−) In this study, no QRDR mutations are reported.

#### 3.2.2 Whole genome sequencing (WGS)

WGS involves DNA extraction, library preparation, sequencing (Illumina/Nanopore), genome assembly, and bioinformatics analysis to detect genetic variants and resistance genes (22). This high-resolution technique enhances epidemiological surveillance and pathogen control by identifying resistance mechanisms and phylogenetic relationships (23).

Of the 27 studies analyzed, only 25.9% (*n* = 7/27) implemented WGS, mostly in poultry isolates. Mutations in QRDR genes were identified in all the studies, with the *gyrA* gene being involved in 71% of the cases. The most frequent mutation in this gene occurred at position 83, reported in 57% of the studies, with changes in various amino acids. On the other hand, mutations in the *parC* gene were observed in 71% of the studies, with the most common being the one at position 57, where in all cases, a threonine to serine change was detected. Regarding PMQR-mediated resistance genes, the *qnrB19* gene was the most reported, present in 43% of the studies. Additionally, different associated plasmid replicons were identified (Table 6).

**Table 6:** Genes conferring resistance to quinolones and fluoroquinolones identified in *Salmonella* spp. using WGS Techniques in South American countries between 2020 and 2024.

Author	Country	Type of sample	Sequence Type (Number of samples)	Gene: Number of mutations	Chromosomal point mutations (QRDR)	PMQR Genes	Plasmids
Saidenberg et al. (37)	Brazil	Asymptomatic broiler chicken feces	*S*. Heidelberg ST15 (*n* = 10) *S. M*innesota ST548 (*n* = 4)	***gyrA***: *n* = 10/10 ***parC***: *n* = 14/14	***gyrA*** (S83F) ***parC*** (T57S)	***qnrB19*** (ColRNAI): *n* = 3/14	ColRNAI, IncX1, IncC, IncI1 y ColpVC, Col156, IncX4, IncFII
Viana et al. (54)		Swines samples from lairage, barn floors, mesenteric lymph nodes, tonsils, swine carcasses and knives	*S*. Typhimurium ST19 (*n* = 16) *S*. I 4,[5],12:i:- ST19 (*n* = 5) *S*. Bredeney ST241 (*n* = 9) *S*. Brandeburgo ST65 (*n* = 4) *S*. Panama ST48 (*n* = 2) *S*. Londres ST155 (*n* = 2) *S. M*bandaka ST413 (*n* = 1) *S*. Derby ST40 (*n* = 1) *S*. Bovismorbificans ST150 (*n* = 1)	***gyrA:*** *n* = 24/41 ***parC***: *n* = 16/41 ***gyrB***: *n* = 1/41	***gyrA*** (D87N) (S83F) (S83Y) ***parC*** (T57S) ***gyrB*** (E466D)	***qnrE1***: *n* = 6/41 ***qnrS1***: *n* = 9/41 ***qnrB19**: n* = 2/41 ***oqxA***: *n* = 2/41 ***oqxB***: *n* = 2/41	ColRNAI, INCr, Incl 1, incA/C2, IncX4, TrfA, IncHI2,inCHI2A, IncFIA(HI1), IncFII(S), IncFIB(S), IncFIC(FII), incY, Col(MGD2), IncFII(Pcry), IncHI1A, IncHI1B(R27),p0111
Vilela et al. (68)		Swine gallbladder, Chicken spleen, gallbladder and illeum	*S*. Choleraesuis ST145 (*n* = 5)	***gyrA***: *n* = 4/5 ***parC:*** *n* = 5/5	***gyrA*** (S83Y) **parC** (T57S)	-	IncX4; IncFIB(S), IncFII(S), IncHI2, IncHI2A, IncFIA(HI1), IncHI1A, IncHI1B(R27), IncFII(S)
Vilela et al. (29)		Chicken meat, bovine meat, animal feed, and a drag swab.	*S.* Heidelberg ST15 (*n* = 11)	***gyrA*** and **parC**: *n* = 11/11	***gyrA*** (S83F) ***parC*** (T57S)	-	ColpVC, IncC, IncX1, and IncI1-I(Alpha)
Benevides et al. (61)		Caecal content laying hens and a laying quail	*S.* Mbandaka ST413 (*n* = 6)	***parC***: *n* = 6/6	***parC*** (T57S)	-	IncHI2A, IncN
Casaux et al. (41)	Uruguay	Calves, cows, heifer, organs, samples from environment, food sample, udder swab, drinking water, bovine fetus autopsy.	*S.* Dublin ST10 (*n* = 6) *S*. Typhimurium ST19 (*n* = 31) *S*. Newport ST45 (*n* = 24) *S.* Anatum ST64 (*n* = 11) *S.* Agona ST13 (*n* = 1) *S.* Montevideo ST138 (*n* = 1) *S*. IIIb 61:i:z53 ST430 (*n* = 1)	***parC***: *n* = 38/75	***parC*** (T57S)	***qnrB19*** (ColE1): *n* = 5/75	IncFII, IncFII(S), IncFIB, IncFIB(S), Col440I, IncI1, IncX1, IncHI2A, IncQ1, IncI2, IncI2(Delta) IncFIC(FII)
Burnett et al. (62)	Ecuador	Poultry	*S*. Infantis ST32 (*n* = 5) *S*. Schwarzengrund ST96 (*n* = 2)	***gyrA***: *n* = 5/7	***gyrA*** (D87Y)	***qnrB19*** (Col440II): *n* = 2/7	IncFIB, Col440II

(−) In this study, no QRDR mutations are reported.

These findings reveal a recurring pattern of specific chromosomal mutations (*gyrA* S83 and *parC* T57) and plasmid-mediated resistance (*qnrB19*) in isolates, which may indicate clonal spread or horizontal gene transfer in the food production chain. The frequent detection of these markers underscores the need for routine WGS-based surveillance in high-risk reservoirs to guide more targeted interventions in antimicrobial resistance control.

**Figure 2:**
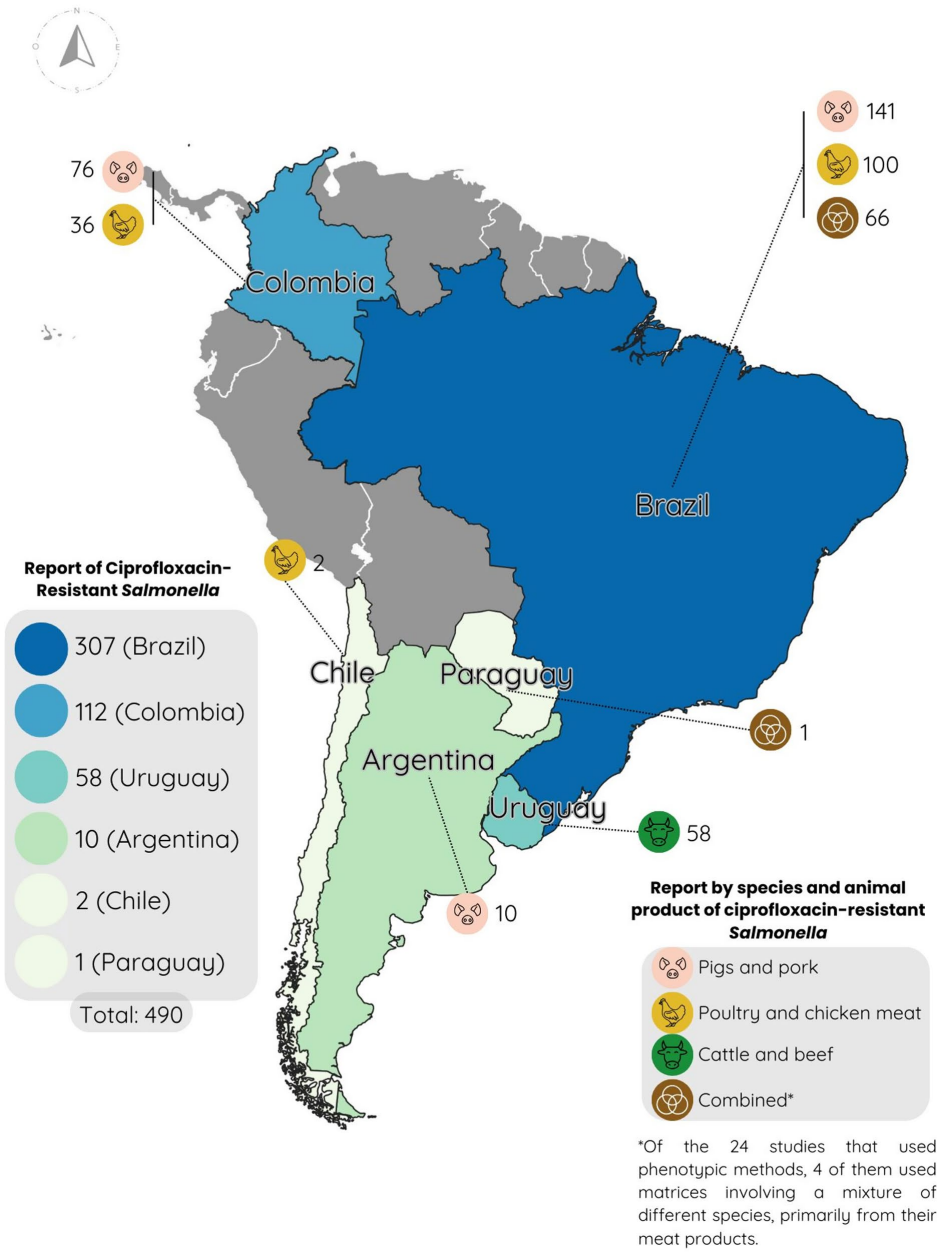
Map of South America with reports of ciprofloxacin resistance percentages reported between 2020 and 2024.

As previously mentioned, resistance to ciprofloxacin is of great importance, as it is the most studied antibiotic in this study and the first-line treatment for both typhoidal and non-typhoidal *Salmonella* spp. infections in humans. Between 2020 and 2024 in South America, a total of 490 *Salmonella* strains were found to be resistant to the antibiotic through phenotypic antibiogram testing, out of 1,781 tested strains, resulting in an overall resistance rate of 27.5%. Specifically, 307 resistant strains were reported in Brazil, 112 in Colombia, 58 in Uruguay, 10 in Argentina, 2 in Chile, and 1 in Paraguay (Figure 2).

## 4 Discussion

### 4.1 Phenotypic resistance and variability between countries and species

Phenotypic resistance to quinolones and fluoroquinolones in *Salmonella* from poultry and swine in South America reveals an alarming trend of antimicrobial resistance that varies between countries and production systems. This is particularly concerning, as fluoroquinolones are classified as “highest priority” by the World Health Organization (WHO) (12), they play a crucial role in the treatment of serious bacterial infections in humans and animals. Their prioritization is due to the fact that, in many cases, they are the only or few therapies available to treat severe non-human-origin infections (24).

Phenotypic resistance in *Salmonella* from poultry fecal samples is widely documented. In Brazil, resistance to nalidixic acid was reported at 90% in cloacal swabs and 28% in other samples, while ciprofloxacin resistance ranged from 0 to 16% (25, 26). In Colombia, resistance to ciprofloxacin (92.3%), levofloxacin (57%), and enrofloxacin (48.7%) was observed in poultry feces (24). Serotypes such as *S. Paratyphi* B, *S*. Minnesota, and *S*. Heidelberg have been identified, with the latter two showing multidrug resistance (MDR) rates of 64–66% (24–26).

In Chile, *Salmonella* isolates from chicken meat show 97% resistance to nalidixic acid, with 94% classified as MDR (27). Similar trends are observed in Brazil, where resistance to nalidixic acid ranges from 94.9 to 100%, likely due to the selective pressure from extensive antibiotic use in poultry production (28, 29). In Brazil, chicken carcass isolates also exhibit high FQ resistence, with ciprofloxacin resistance reaching 81.82% and MDR rates at 63.64% (30). However, other studies report significantly lower ciprofloxacin resistance (0.93–2.08%), suggesting possible methodological differences or evolving resistance patterns over time (31, 32).

In Colombia, older studies reported low resistance in poultry farms to levofloxacin, 2.3% in Cundinamarca, 0% in Santander (33), but more recent data show a sharp increase in resistance, likely due to continued antibiotic use (24). Genetic studies highlight *Salmonella* Heidelberg (ST15) as a major concern due to its high morbidity, resistance, and outbreak potential, posing a significant public health risk (34–37).

### 4.2 Genotypic resistance and key mutations

Genotypic studies identified key mutations in *gyrA* and *parC*, along with the *qnrB19* gene, as major contributors to fluoroquinolone resistance (34, 35). Many ciprofloxacin-resistant strains carried both *gyrA* mutations and the *qnrB* gene, enhancing resistance (38). A study on *S*. Heidelberg from Brazilian poultry meat imported to the Netherlands found *parC* mutations in all 122 isolates, with 96.7% also carrying *gyrA* mutations (39). Similarly, *qnrB19* was detected in *S.* Minnesota from Brazilian poultry meat (35, 37). The *aac(6′)-Ib-cr* gene, linked to fluoroquinolone resistance, was found in one *S.* Heidelberg isolate from Colombian broiler farms, which showed resistance to ciprofloxacin and levofloxacin (24, 40).

Antimicrobial resistance in *Salmonella* from swine varies across countries, influenced by production practices, antibiotic use, and regulations. In Brazil, de Quadros et al. (63) found that only 16% of *Salmonella* strains were fully susceptible, yet enrofloxacin inhibited 100% of them. This is notable, as enrofloxacin was widely used before 2017. However, stricter fluoroquinolone regulations in pork production appear to have reduced resistance, indicating a positive impact of recent policies (41).

However, Kich et al. (64) reported high nalidixic acid resistance (45.9%) in carcasses, while studies on mesenteric lymph nodes found resistance rates of 40.6–65.5% and MDR rates of 70.3–79% (42, 43). In Argentina, Vico et al. (66) observed 52% resistance to fluoroquinolones in mesenteric lymph nodes and 86% MDR. Resistance in *S. Typhimurium* and *S*. Derby, a key serotype in swine and pork products worldwide, is particularly concerning (44–46). The role of *S*. Derby, which is one of the most frequently reported serotypes in swine and pork products, both in Brazil and other regions such as the European Union and China (46, 47). In Colombia, Vidal et al. (67) reported 44% MDR in *Salmonella* from swine feces, with 55% ciprofloxacin resistance, emphasizing the impact of antibiotic overuse in pig farming.

Recent studies reveal distinct resistance patterns between poultry and swine. Pigs show higher resistance to azithromycin, ampicillin, and chloramphenicol, while poultry exhibit greater resistance to quinolones and sulfonamides, reflecting differences in antibiotic use between both industries (48).

Antimicrobial resistance data for cattle is limited. In Uruguay, high non-susceptibility to FQ has been reported, with 77.3% of isolates non-susceptible to ciprofloxacin and 6.6% to enrofloxacin (41). Additionally, 56% of isolates exhibited MDR particularly in serotypes like *S. Typhimurium*, *S*. Newport, and *S*. Anatum are notably prevalent in intensive cattle production, causing both enteric and invasive diseases such as septicemia (49).

Genotypic studies identify ST19 (*S. Typhimurium*) and its monophasic variant as key serovars in swine, commonly detected in Brazil and the European Union (50–54). *S. Typhimurium* is the second most common cause of salmonellosis outbreaks in the EU, with pork as the main source in 2014 (55).

The Ser83Phe mutation is frequently reported in pigs strains with reduced susceptibility to CIP, which has been detected in clinical cases in Peru (56). PMQR genes of the *qnr* alleles, such as the *qnrB19* gene, are associated with the spread of *Salmonella* strains resistant to CIP in the United States (57). The Thr57Ser mutation has been described as relevant for reducing susceptibility to ciprofloxacin (58). The frequent detection of *gyrA* (position 83) and *parC* (position 57) mutations highlights their critical role in fluoroquinolone resistance. These findings underscore the need for targeted surveillance and intervention strategies to mitigate the spread of resistant strains.

### 4.3 One Health implications and need for coordinated surveillance

The antimicrobial resistance observed to quinolones and fluoroquinolones reflects the selective pressure generated by the use of antibiotics in intensive animal production systems. This resistance can vary considerably between different matrices, such as meat, feces and carcass swabs, highlighting the importance of sampling multiple sources within the production system. Although antibiotics remain a crucial tool for treating bacterial diseases, the increase in antimicrobial resistance has reduced their effectiveness (59). Resistance to fluoroquinolones is particularly concerning, as they are commonly used to treat severe human infections, such as systemic salmonellosis in immunocompromised individuals (60).

These findings underscore the urgency of adopting a coordinated One Health approach that integrates surveillance, policy, and intervention strategies across human, animal, and environmental sectors to effectively mitigate the spread of antimicrobial resistance in South America.

### 4.4 Limitations

This study has several limitations that should be considered when interpreting the results. First, not all South American countries published research on quinolone- and fluoroquinolone-resistant *Salmonella* between 2020 and 2024, which limits the representativeness of the data for the entire region. In addition, there was considerable variability in the types of samples or matrices used in the included studies—ranging from feces, tissues, food, and in some cases pooled samples from different animal species—making it difficult to isolate *Salmonella*-specific information.

Although the study initially sought to include data on fluoroquinolone-resistant *Salmonella* from rodents, no eligible studies were found from South America during the selected period. However, in veterinary medicine, rodents are recognized as important reservoirs and amplifiers of zoonotic infections, including *Salmonella*. Their absence in the published literature highlights a relevant knowledge gap that should be addressed in future research.

Finally, although a predefined protocol was used to guide the review process, it was not formally registered in a database such as PROSPERO. This omission is acknowledged as a limitation in the transparency of the study methodology.

Future studies should aim to include a broader range of countries and matrices, and explore the role of rodents in the dissemination of antimicrobial-resistant *Salmonella*.

## 5 Conclusion

The antimicrobial resistance to quinolones and fluoroquinolones in *Salmonella* from poultry, swine, and cattle in South America shows a concerning trend, with high rates of resistance and multidrug resistance across several countries. The indiscriminate use of antibiotics in animal production systems appears to be a key factor in the accelerating pressure of the problem, which could undermine the effectiveness of treatments in both humans and animals. Furthermore, resistance varies significantly across different matrices, highlighting the need for more comprehensive surveillance and the use of more standardized diagnostic techniques. The findings of this study highlight the urgent need for coordinated regional efforts to monitor and control antimicrobial resistance in *Salmonella.* Policymakers, researchers, and industry stakeholders must collaborate to implement effective strategies that safeguard public health and ensure food safety.
